# From Biology to Clinical Practice: Iron Chelation Therapy With Deferasirox

**DOI:** 10.3389/fonc.2021.752192

**Published:** 2021-10-06

**Authors:** Giuseppe A. Palumbo, Sara Galimberti, Wilma Barcellini, Daniela Cilloni, Nicola Di Renzo, Elena Maria Elli, Carlo Finelli, Luca Maurillo, Alessandra Ricco, Pellegrino Musto, Rodolfo Russo, Roberto Latagliata

**Affiliations:** ^1^ Department of Scienze Mediche Chirurgiche e Tecnologie Avanzate “G.F. Ingrassia, ” University of Catania, Catania, Italy; ^2^ Section of Hematology, Department of Clinical and Experimental Medicine, University of Pisa, Pisa, Italy; ^3^ Hematology, Fondazione Istituto di Ricovero e Cura a Carattere Scientifico (IRCCS) Ca’ Granda Ospedale Maggiore Policlinico di Milano and University of Milan, Milan, Italy; ^4^ Department of Clinical and Biological Sciences, University of Turin, Turin, Italy; ^5^ Hematology and Transplant Unit, Ospedale Vito Fazzi, Lecce, Italy; ^6^ Division of Hematology and Bone Marrow Unit, Ospedale San Gerardo, Aziende Socio Sanitarie Territoriali (ASST), Monza, Italy; ^7^ IRCCS Azienda Ospedaliero-Universitaria di Bologna, Istituto di Ematologia “Seràgnoli”, Bologna, Italy; ^8^ Department of Onco-hematology, Fondazione Policlinico Tor Vergata, Rome, Italy; ^9^ Unit of Hematology and Stem Cell Transplantation, Azienda Ospedaliera Universitaria (AOU) Consorziale Policlinico, Bari, Italy; ^10^ Department of Emergency and Organ Transplantation, “Aldo Moro” University School of Medicine, Bari, Italy; ^11^ Clinica Nefrologica, Dialisi e Trapianto, Department of Integrated Medicine with the Territory, IRCCS Ospedale Policlinico San Martino, Genoa, Italy; ^12^ Unità Operativa Complessa (UOC) Ematologia, Ospedale Belcolle, Viterbo and Division of Cellular Biotechnology and Hematology, Sapienza University, Rome, Italy

**Keywords:** iron chelation therapy (ICT), deferasirox, myelodysplastic syndromes (MDS), myelofibrosis (MF), radical oxygen species (ROS), iron toxicity, anemia

## Abstract

Iron chelation therapy (ICT) has become a mainstay in heavily transfused hematological patients, with the aim to reduce iron overload (IOL) and prevent organ damage. This therapeutic approach is already widely used in thalassemic patients and in low-risk Myelodysplastic Syndrome (MDS) patients. More recently, ICT has been proposed for high-risk MDS, especially when an allogeneic bone marrow transplantation has been planned. Furthermore, other hematological and hereditary disorders, characterized by considerable transfusion support to manage anemia, could benefit from this therapy. Meanwhile, data accumulated on how iron toxicity could exacerbate anemia and other clinical comorbidities due to oxidative stress radical oxygen species (ROS) mediated by free iron species. Taking all into consideration, together with the availability of approved oral iron chelators, we envision a larger use of ICT in the near future. The aim of this review is to better identify those non-thalassemic patients who can benefit from ICT and give practical tips for management of this therapeutic strategy.

## Introduction

Iron chelation therapy (ICT) has become a mainstay in heavily transfused hematological patients, with the aim to reduce iron overload (IOL) and prevent organ damage. International and Italian guidelines recommend ICT in low-risk Myelodysplastic Syndrome (MDS) patients (1). ICT should also be considered for transfusion-dependent patients with high-risk disease, when they are responding to therapies able to modify their life expectancy or if they are candidate for an allogeneic stem cell transplantation (HSCT) in their therapeutic program (2, 3).

Considering hematological and hereditary disorders, characterized by considerable transfusion support to manage anemia, consequent iron toxicity contributes to exacerbate anemia and other clinical comorbidities due to oxidative stress radical oxygen species (ROS) mediated by free iron species. Therefore, ICT has been introduced in the last years also in these additional categories of patients recognizing the importance of this therapy beyond the supportive aim, to counteract iron toxicity itself.

Approved iron chelators in the abovementioned diseases are deferoxamine (subcutaneous route) and deferasirox (oral administration). Unlike thalassemic patients, in whom it should be continued indefinitely, in patients with MDS and other hematological conditions, ICT can usually be continued until resolution of transfusion dependency and/or normalization of iron toxicity available markers (4–8).

## The Current Role of Iron Chelation Therapy in Myelodysplastic Syndromes

### Why to Use ICT in Patients With MDS?

#### Mechanisms That Can Favor Iron Toxicity in Patients With MDS

MDSs belong to a family of clonal dysfunctions of the hematopoietic system, which result in failure of bone marrow hematopoiesis (ineffective hematopoiesis) and in an increased risk of progression to acute myeloid leukemia (AML). A common complication observed in these anemic patients is iron toxicity, which not only causes organ damage in liver, heart, and endocrine glands but also has toxic effects on the bone marrow niche, favoring ineffective hematopoiesis, genomic instability, and eventual clonal evolution towards acute leukemia, with consequent negative implications for survival and quality of life (9, 10).

Dyserythropoiesis affects the regulation of iron homeostasis, favoring increased intestinal absorption and plasma recirculation. In this setting, the two key factors involved in the mechanisms triggered by ineffective erythropoiesis are

- hepcidin, whose generally decreased levels vary in the different forms of MDS, with lower values observed in lower-risk MDS and higher values in higher-risk MDS and in chronic myelomonocytic leukemia (CMML) (11)
- hypoxia, which increases the expression of DMT1, DCYTB, and FPN in the enterocytes, the epithelial cells that form the epithelial lining of the intestinal villi, resulting in increased intestinal iron absorption (12).

These pathological iron load mechanisms are active regardless of the need for transfusions, although transfusion support considerably worsens this pre-existing overload (9, 13–15). Indeed, each unit of packed red cells contains 200–250 mg of iron (approximately 100 times the normal daily intake). Moreover, at diagnosis 80% of patients with MDS present already anemia due to ineffective erythropoiesis and requiring blood transfusions (16–18). Consequently, it is very common that chronic transfusion-dependent patients will develop IOL.

#### Definition of a Genetic Profile Predisposing for Iron Overload

Different gene mutations and/or alterations that have a direct effect on iron metabolism and accumulation have been reported in certain forms of MDS. These include the following:
- SF3B1 mutations: these mutations, frequently observed in MDS patients and especially when ring sideroblasts (RS) are present, dysregulate the RNA splicing of the erythroid transcription factors TAL1 and GATA1, resulting in increased but ineffective erythropoiesis (19). SF3B1-mutated patients present mitochondrial iron accumulation (20) and increased expression of a specific isoform of SLC25A37 (an important transporter of Fe(2+) into the mitochondria) (21). Splicing alterations have been observed in the key genes associated with iron accumulation.
- 5q deletion: this karyotypic alteration results in considerable ineffective erythropoiesis associated with the heterozygous deletion of RPS14. RPS14 haploinsufficiency activates the innate immune system and increases the expression of S100A8-S100A9, resulting in p53-dependent erythroid differentiation defects (22).
- TET2 mutations: (only in a subset of patients with MDS) may be involved in iron metabolism and in heme biosynthesis in the erythroblasts. Studies using TET2 knockdown mouse models have shown high serum and mitochondrial ferritin levels and dysregulation in a number of genes involved in iron metabolism (23).

#### Diagnostic Workup for Iron Toxicity in Patients With MDS

The approaches currently employed to diagnose iron overload include

- estimate iron intake following transfusions;
- serum ferritin and transferrin saturation measurements (blood ferritin levels must be monitored monthly, whereas follow-up monitoring of the parameter trends should be performed every 3–6 months);
- magnetic resonance imaging (MRI) of liver, pancreas and heart evaluation when available
- morphological evaluation of bone marrow iron by Perls staining.

Serum ferritin is an indirect and unreliable measurement of the entity of iron deposits in the body; although there is no validated threshold, as for other conditions such as thalassemia, experts consider a ferritin level >1,000 ng/ml to be suggestive of IOL and suggest serial/periodic evaluation of ferritin to evaluate trends instead of absolute values. However, as high ferritin levels may be secondary to an inflammatory state regardless of iron burden, it is always advisable to assess ferritin trends and transferrin saturation suggesting the presence of unbound iron that is harmful for several organs (24).

Transferrin saturation values >60–70% are correlated with free iron in plasma as non-transferrin-bound iron (NTBI) (5, 25–27). A subcomponent of NTBI, called labile plasma iron (LPI), is a potent redox-active form capable of permeating cells through free cellular ionic channels, inducing enlargement of the amount of labile cellular iron (LCI) that in turns leads to increased levels of intracellular ROS. The consequent intracellular oxidative stress condition cause iron-related cellular damages or even cellular death (10).

Unfortunately, at the time being, measurements of NTBI and LPI are not yet standardized methods, available only in selected research laboratories.

### Lower-Risk MDS Patients: How and When to Chelate

#### When to Start Iron Chelation Therapy

Current guidelines (SIE, ELN, NCCN) (2, 28, 29) consider the following parameters as criteria for the initiation of ICT in patients with lower-risk MDS:

- Transfusion dependency (with transfusion burden of at least 20 units of packed red cells).
- Ferritin level >1,000 ng/ml.
- Life expectancy >12–24 months.
- When the chosen drug is deferasirox, CrCl has to be >60 ml/min; the starting dose of deferasirox is usually 7 mg/kg for the new formulation.

Evidence of oxidative stress due to iron-induced ROS before the conventional limit of 20 transfused units might lead to review current indications for early starting ICT providing a better tolerated lower-dose treatment (for deferasirox, 3.5 mg/kg) for reducing iron overload and thus ROS overproduction promptly (1).

Hence, it is under discussion the ideal approach to candidate patients which might lead to an expansion of the indications for ICT, including the following:

- Patients with transfusion-dependent MDS (and bone marrow failure in general), regardless of the International Prognostic Scoring System (IPSS) risk level, taking into consideration the presumable life expectancy
- Patients with a transfusion history (even if recently started) and/or ferritin ≥500 ng/ml, in the absence of hepatocellular necrosis and/or inflammation

#### When to Discontinue Iron Chelation Therapy

Although there is currently a divergent opinion on the optimal duration of ICT, some guidelines (NCCN, ELN) (28, 29) recommend considering therapy interruption when serum ferritin levels are <1,000 ng/ml (whereas in the SmPc for deferasirox, the threshold value is <500 ng/ml). A consensus statement issued by the MDS Foundation Working Group in 2008 suggests continuing therapy for the entire duration of transfusion need (1). With the aim to prevent damage induced by free toxic iron exposure rather than iron bulk, it might be useful to consider not only ferritin values but also transferrin saturation, when considering the possibility of discontinuing the chelation treatment. Although the threshold value of transferrin saturation that protects from organ damage is not yet known, the iron chelation optimal goal should be a normal transferrin saturation value. Finally, when ferritin levels during deferasirox therapy goes below 300 µg/L, a temporary discontinuation should be taken into consideration for a potential toxicity increase of chelation therapy itself.

### Clinical Outcomes and Iron Chelation With Deferasirox in Lower-Risk MDS

In **Table 1**, response to chelation treatment, rate of discontinuation, and erythroid response observed in the main studies with DFX in MDS low-risk setting are reported.

**Table 1:** Response to chelation treatment, rate of discontinuation, and erythroid response in the main studies with deferasirox in MDS low-risk setting.

	N° Pts	Dosage (mg/kg/day)	Ferritin at baseline, ng/ml (median)	Ferritin at 12^th^ month, ng/ml (median)	p	% stop DFX at 12^th^ month	Erythroid response (%)
**EPIC** (30)	341	10–30	2,730	1,904	0.002	48.7	22.6
**GIMEMA** **MDS0306** (31)	152	10–20	1,966	1,475	<0.0001	55.2	15.5
**GERMAN** **STUDY** (32)	50	20–30	2,447	1,685	0.01	52.0	11.0
**US** **STUDY** (33)	176	20	2,771	2,210	<0.001	47.0	15.0
**Hematology** **Sapienza** (34)	40	10–30	2,878	1,400	<0.001	40.0	10.0
**GROM +** **Basilicata** (35)	118	10–20	1,773	1,300	<0.001	35.0	19.0
**GRUPPO** **CAMPANO** (36)	55	10–30	2,362	683	0.001	NR	29
**STUDY** **EXTEND** (37)	123	10–20	2,679	2,000	0.0002	37.7	NR
**STUDY** **EXJANGE** (37)	44	10–30	2,442	2,077	0.06	29.5	NR

#### Possible Effects of Iron Chelation in Addition to the Decrease in Iron Overload

In MDS patients, when assessing iron-induced damage, changes in the dynamic equilibrium between functional iron pool and deposited iron pool must be considered in addition to tissue iron concentrations.

This deposition results in exposure to iron, which causes organ damage mainly to the heart, blood vessels, and bone marrow, a worsening hematopoiesis, and an enhanced clonal instability.

**Figure 1:**
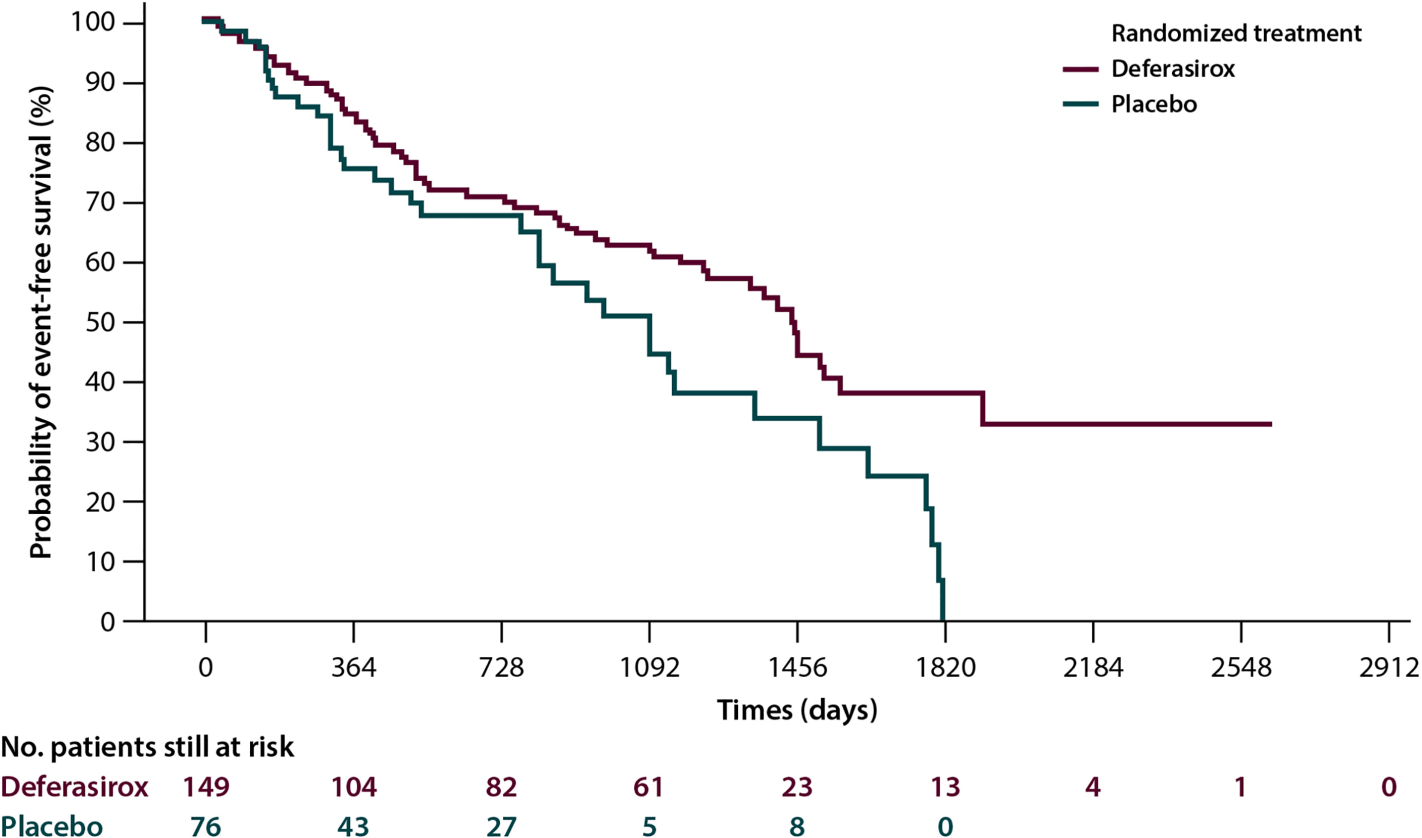
Event-free survival in the Telesto trial according to treatment arm, and 225 myelodysplastic patients were included, 149 in the deferasirox arm and 76 in the placebo one (42).

**Figure 2:**
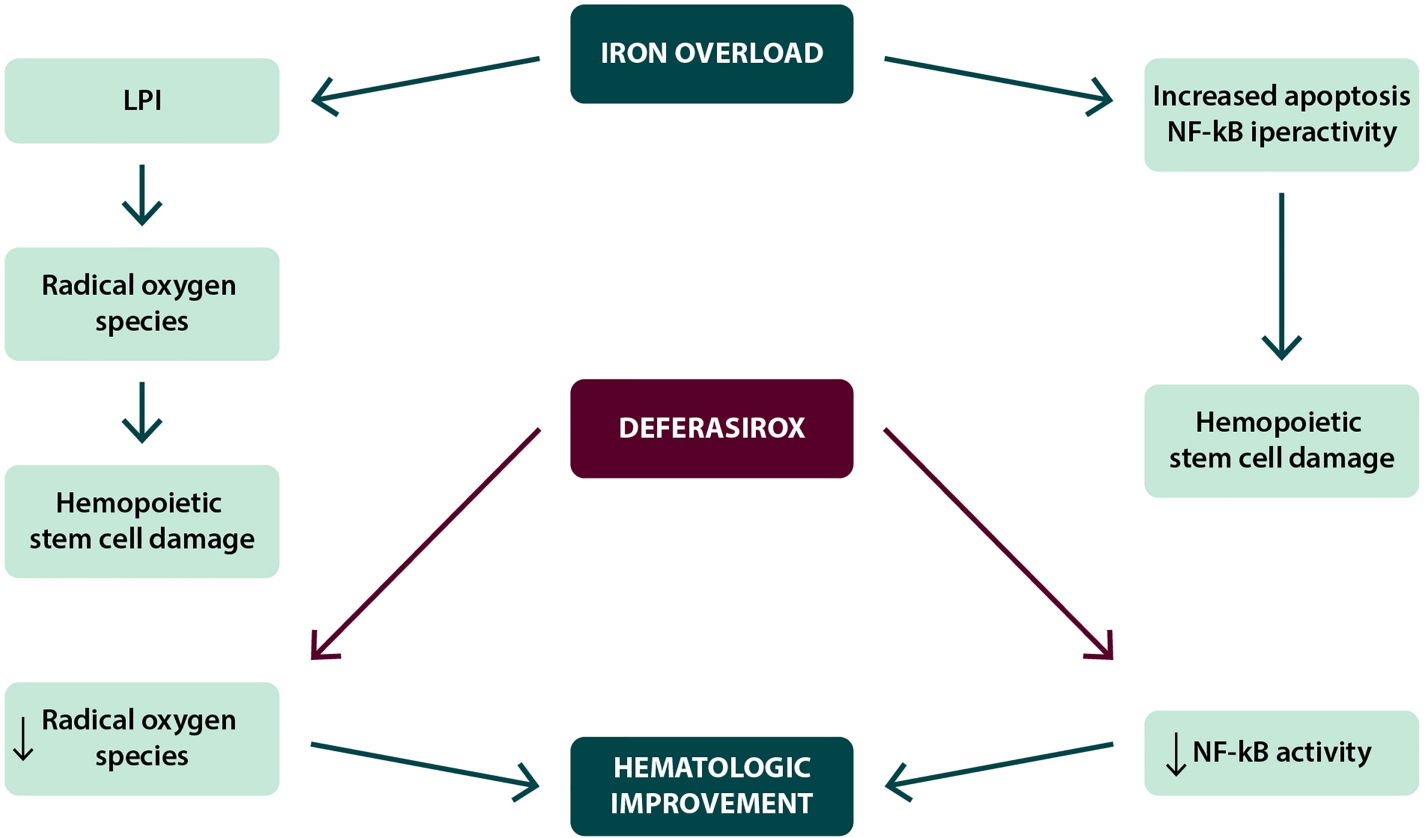
Possible mechanisms in the achievement of hematological improvement during deferasirox treatment.

In MDS, further clinical outcomes are observed during chelation treatment with deferasirox, such as the following:

- *Overall survival (OS).* A number of retrospective studies show that chelated MDS patients have a better median survival than those who are not chelated. This finding was subsequently confirmed by a meta-analysis (eight studies for a total of 1,562 patients), which reported an advantage in terms of survival greater than 61.2 months (38). Although these observations may be partly attributed to selected bias, the finding of a positive effect of ICT on the OS of patients with MDS would appear to be fairly consistent. In addition, a Canadian prospective study that collected data from March 2006 to July 2016 demonstrated the advantage in terms of survival in patients with MDS receiving ICT independently by confounding factors such as age, comorbidities, the Revised International Prognostic Scoring System (R-IPSS), and treatment with disease-modifying drugs (39). An improvement in OS for ICT patients was also evidenced in a propensity-score analysis on transfused lower-risk MDS subjects in the European MDS Registry (40). In addition to an increase in OS (133 *versus* 105 months; p=0.009), the IRON2 retrospective study reported a significant increase in cardiac event-free survival (EFS) in the 146 patients who received ICT (of whom 72% treated with deferasirox) compared to those who did not (137 *versus* 90 months; p=0.004) (41).Focusing on the clinical outcomes, the results of the TELESTO randomized, double-blind prospective study, which evaluated the effects of treatment with deferasirox *versus* placebo in patients with low-/int-1-risk MDS have been reported. The primary endpoint of the study was EFS: of a total of 225 randomized patients, the group treated with deferasirox showed a median EFS (time to the first adverse event, constituted by clinically significant organ toxicity, including cardiac and hepatic events, evolution to leukemia or death) that was significantly longer than that observed in the placebo arm (1,440 *vs.*1,091 days; risk reduction: 36.4; p=0.015) (**Figure 1**). The use of this molecule is therefore supported in patients with low-/int-1-risk MDS with iron overload (42).
- *Evolution to acute leukemia.* Regarding the risk of evolution to leukemia, data are still controversial: preclinical models describe the effect of ICT to reduce genetic instability induced by the greater production of ROS. Indeed, a significant reduction in leukemia-free survival (LFS) has been reported in patients with transfusion-dependent disease (41, 43). The American US22 registry data on 600 patients with transfusion-dependent low-risk MDS [of whom 263 received ICT, the vast majority with deferasirox (44)] provided prospective evidence that ICT has a positive effect. In addition to OS, in this cohort, LFS was also better amongst patients receiving ICT, with a median time to progression of 40.6 *vs* 27.3 months.Similar results were achieved in an observational study on 97 patients with low-/intermediate-risk MDS, with a trend towards shorter LFS in patients who did not receive ICT: at 30 months, 34% of patients not treated with an iron chelator had progressed to AML *vs* 17% of patients receiving iron chelators (45).
- *Hematological improvement.* In several clinical studies, including the EPIC (Evaluation of Patients’ Iron Chelation with Exjade) study, a percentage of patients (10–20%) treated with deferasirox obtained a positive effect on hematopoiesis, with erythroid hematological recovery (30–33), as reported in **Table 1**. Similar results were also observed in Italian and international real-world studies. In the data reported by two Italian regional registries, the Roman Myelodysplasia Group (GROM) and the Basilicata Registry, including a total of 118 patients with transfusion-dependent MDS treated with deferasirox, hematologic improvements in the erythroid, platelet, and neutrophil series were reported in 17.6, 5.9, and 7.1% of cases, respectively (35). In the prospective randomized TELESTO study, 27/127 patients (22.3%) receiving deferasirox obtained hematological improvement; however, it is worth of note that a similar rate of hematological improvement (14/68 patients, 20.6%) was also observed in the placebo arm of the study (42). Although several mechanisms have been postulated, the reduced production of iron-dependent ROS and inhibition of NFk-B activity would appear to be particularly relevant factors (**Figure 2**).

### Higher-Risk MDS Patients: How and When to Chelate

According to prognostic scores such as the IPSS, R-IPSS, and WHO Prognostic Scoring System (WPSS), patients with “high-risk” MDS account for 20–30% of the total population. Historically, life expectancy in this group of patients has been reported between 1 and 2 years; however, the introduction of disease-modifying therapies (such as hypomethylating agents) has led to longer survival and better prognosis (16).

The main causes of death in two-thirds of patients with high-risk MDS are strongly correlated with cardiac events and infectious complications (greater risk in transfused patients than in those who do not receive transfusions), at least partly due to the impact of iron-induced toxicity (46).

The main limiting factors to start ICT are the short OS of these patients, the potential higher risk of renal or hepatic impairment and gastrointestinal bleeding.

The data currently available are constituted by a retrospective study on 51 patients with transfusion-dependent intermediate-/high-risk MDS (in 71% azacitidine was co-administered). Treatment with deferasirox in these patients showed a significant clinical benefit in terms of decrease in ferritin levels, liver marker normalization, and (in one case) hematological improvement, without significant additional toxicity (median follow-up was 35.3 months, and median OS 37.5 months) (3). Some registries (US Medicare, SEER) that already reported a higher incidence of infections in transfused than in non-transfused MDS patients (45–47) reported data on deferasirox treatment able to reduce risk of infection in patients more likely suffering these events for iron toxicity exposure due to transfusion. Prospective studies evaluating the combination of azazitidine and deferasirox in HR-MDS are ongoing.

In patients with high-risk MDS, there is also new evidence of a significant effect in terms of genotoxicity accumulation of IOL-induced mutations, which might add a biological strong rationale for the administration of ICT. *In vivo* evidence also shows that oxidative stress may contribute to the hypermethylation of important tumor suppressor genes, suggesting a synergic action of ICT and treatment with hypomethylating agents (48).

The ideal candidates for ICT amongst patients with higher-risk MDS are those with a longer life expectancy and more favorable prognostic factors, including

- good general health and age ≤65 years,
- absence of significant comorbidities, and
- patients who are candidates for allogeneic transplantation and/or treatment with demethylating agents.

#### When to Start Iron Chelation Therapy and When to Discontinue Iron Chelation Therapy

The possible parameters for starting ICT in patients with high-risk MDS are the same as described previously for lower-risk patients. Decisions regarding the duration of the treatment should also be based on the same criteria already described.

### Efficacy and Safety of Deferasirox in Patients With MDS: How to Administer Iron Chelation Therapy

#### How to Measure the Efficacy of Iron Chelation Therapy in Terms of the Reduction in Iron Overload in Patients With MDS

The assessment of the efficacy of the treatment may be based on two types of parameters:

1. Iron toxicity markers
2. Patient clinical outcome

The biochemical and tissue damage markers that indicate a reduction in IOL and constitute the parameters for the administration of deferasirox are

- transferrin saturation,
- serum ferritin,
- aminotransferases (a decrease in aminotransferase with improvement of liver function has been observed in a significant proportion of patients with abnormal values at baseline),
- measurement of labile plasma iron (LPI), where available, and
- T2-weighted MRI (where available).

In the published clinical studies, serum ferritin levels serially measured throughout the observation period were the main used parameters (16, 49).

Clinical outcomes that can be directly measured and therefore compared, alongside cardiac events, infectious complications, and disease progression, were hematologic response rates, in terms of trilinear improvement (erythroid, platelet, and neutrophil compartment). The relationship between these parameters and the reduction in the markers of IOL was significant in some cases, but not in all (46).

Any kind of hematologic responses, which was observed in 20% of patients treated with deferasirox, can be considered an additional clinical benefit constituting a further reason for continuing ICT, and its absence does not constitute an indication for discontinuing treatment and it does not, therefore, represent a criterion for a therapeutic decision-making.

#### How to Monitor the Safety of the Iron Chelation Therapy

The most important studies in MDS setting reported data on old formulation deferasirox safety profile, and the most common side effects were gastrointestinal (GI) ones due to lactose; in particular, diarrhea, nausea, and abdominal pain could occur. The safety profile of deferasirox’s new formulation, now available in film-coated tablets, was studied in ECLIPSE trial: the incidence and type of adverse events (AEs) for the new formulation are similar to the previous one (tablets for oral suspension), whereas the incidence of severe AEs (SAE) is lower (19.5 *versus* 25.6%), especially for GI events such as diarrhea, nausea, and vomiting (50). The study also reports the same similarity also for hepatic and renal safety (the CrCl values to start treatment remained ≥60 ml/min). Patients receiving treatment with deferasirox can be subjected to the same safety monitoring scheme.

#### Guidance to be Given to Patients on Iron Chelation Therapy

It is essential to provide patients with clear and complete information concerning the main aspects of ICT. These aspects regard

- treatment compliance, which is extremely important for the efficacy of therapy, as the better treatment compliance observed amongst patients treated with the new formulation (92.9 *vs.* 85.3%) coincided with a greater decrease in ferritin levels in this group (median: –14% vs. –4.1%) (50); and
- safety management: it is important to instruct patients how to manage adverse events and eventually refer them promptly to their physician.

Hence, patient education can improve compliance with ICT and reduce the complications associated with IOL (51).

## Iron Chelation Therapy in Myelofibrosis and Other Chronic Anemias

### Pathogenesis and Clinical Significance of Anemia in Myelofibrosis

#### The Pathogenetic Mechanisms of Anemia in Myelofibrosis 

Myelofibrosis (MF) is a rare hematological condition classified in the group of chronic BCR-ABL1- negative myeloproliferative neoplasms (MPN) (52), characterized by bone marrow fibrosis and consequent ineffective hematopoiesis, with the onset of extramedullary hematopoiesis. The signs and symptoms characterizing this condition include

- constitutional symptoms (itching, fever, night sweats and weight loss);
- symptoms associated with splenomegaly;
- symptoms due to anemia (asthenia, dyspnea, palpitations) (53): huge symptom burden, poorer quality of life, and iron overload associated with transfusion therapy. As bone marrow fibrosis, anemia increases progressively over time from 38% of cases with Hb <10 g/dl at diagnosis to 64% 1 year after diagnosis (54).

Although anemia is caused by bone marrow erythropoietic tissue fibrotic replacement and by ineffective production and maturation of red blood cells generated by a compensatory extramedullary erythropoiesis, these are not the only etiological factors. Other significant pathogenetic mechanisms causing anemia are splenic sequestration and hemodilution, resulting from the increase in plasma volume and abnormal production of bone marrow cytokines. As a consequence, MF is characterized by a considerable local and systemic inflammatory state, with a wide increase of inflammatory bone marrow cytokines, which in turn affect erythropoiesis in the residual functional areas of hematopoietic bone marrow. This pro-inflammatory state includes an increase in circulating hepcidin levels interfering with iron metabolism as observed in other secondary anemias (55).

MF patients present specific genetic changes too likely anemia-related: patients with Calreticulin (CALR) or MPL thrombopoietin receptor (MPL) gene mutations would appear to have a lower likelihood of developing anemia than triple-negative patients (CALR, MPL, and JAK2 wild types), with underlying unknown mechanisms at this time.

The presence of anemia is a negative variable recognized in all the scores that stratify MF patient risk from a prognostic standpoint (56), with a negative impact on life expectancy (by stratifying patients with primary MF according to the degree of anemia, we observe median OS values that range from 7.9 years in patients without anemia to 2.1 years in cases with severe anemia) (57).

Anemia and thrombocytopenia in patients with MF could worsen with treatment with ruxolitinib, the first Janus Kinase 1/2 (JAK) inhibitor approved on the basis of the COMFORT and JUMP study results (58, 59). Ruxolitinib acts directly on the hyperactivation of the JAK-STAT pathway that underlies the disease, by selectively inhibiting the JAK-1 and JAK-2 kinases and suppressing residual bone marrow function (55). The anemia due to ruxolitinib treatment has not to be considered as negative prognostic factor, as it is often transient and does not worsen the patient’s overall outcome, unlike pre-existing anemia. Most cases of anemia during ruxolitinib therapy appear within the first 6 months and are Grade 1/2, rarely requiring the discontinuation of therapy. In general, it is advisable to strictly monitor patients during the first weeks of therapy when the Hb levels could decrease rapidly to promptly evaluate the possible transfusion need, before increasing again in the following months, and stabilize on average approximately 10% below the baseline value (60).

The onset of anemia does not reduce the efficacy of ruxolitinib on splenomegaly and symptoms (58, 61), and consequently treatment is strongly recommended in anemic patients too. Similarly, it is not advisable to reduce the dose of ruxolitinib in patients who develop anemia, as this side effect seems not dose-dependent: the treatment should be continued at the doses required to control the disease and established on the basis of the platelet count.

Therefore, three types of patients can be identified:

- Patients who are not transfusion-dependent at baseline
- Patients who are already transfusion-dependent at baseline and who usually remain so throughout treatment
- Patients just above the dependency threshold at baseline, who become transfusion-dependent after therapy (these borderline patients can be the most difficult to manage, as they are not used to receiving transfusion support)

### Iron Toxicity and Transfusion Need in Myelofibrosis

#### ­The Pathogenic Mechanisms of Oxidative Stress Iron-Mediated in Myelofibrosis and Organ Toxicity

The main treatment for anemia consists of RBC transfusions: approximately a quarter of patients with MF are transfusion-dependent at diagnosis (when dependency is defined as the administration of >6 units of RBC over a period of 12 weeks for Hb levels <8.5 g/dl), and many more become so during the course of their illness (55).

In patients with MF, characterized by a significant pro-inflammatory state, hepcidin levels are usually higher, with a consequent unbalance of iron metabolism and showing a negative prognostic value (62).

Although the vascular effects of free iron species have been less extensively studied, it has been observed that high circulating iron levels can worsen the atherosclerotic phenotype, especially in elderly patients: the macrophages on the vessel wall accumulate the iron produced by the increased destruction of red blood cells and abnormal iron homeostasis, with an increase in the production of ROS and decreased cholesterol outflow. The resulting oxidative stress promotes the formation of foamy cells, inflammation, apoptosis, and the deterioration of the atherosclerotic plaque. Furthermore, the high hepcidin levels typical of MF contribute to this process by blocking ferroportin and, therefore, the exportation of iron from plaque (63).

ICT can have a beneficial effect on iron-mediated endothelial dysfunction, because the binding of the labile cell iron in the vessel wall may reduce the formation of ROS and the inactivation of nitric oxide, another consequence of iron-induced toxicity. It was observed that deferasirox can significantly improve the dilation of the brachial artery and reduce carotid artery stiffness in patients with beta-thalassemia (64).

In bone marrow, IOL and the consequent oxidative stress cause an increase of genetic instability of the hematopoietic cells and the production of pro-inflammatory cytokines by bone marrow stromal cells that, in turn, may play a role on bone marrow failure in patients with MF, as it was reported in MDS (15). However, iron-induced toxicity and its negative impact on the hematopoietic microenvironment (65) might have an impact on the dysregulation of the JAK-STAT pathway itself, which is able to induce an increase in pro-inflammatory and fibrogenic cytokines and increased ROS production. In addition, patients with MF present significant NF-kB pathway activation, which induces the production of inflammatory cytokines (IL6, TNFα) and interacts with the dysregulation of the JAK-STAT pathway to amplify the oxidative damage (66, 67).

#### Assessment and Monitoring of Iron-Induced Toxicity in Patients With Myelofibrosis

As for other conditions ferritin remains the main parameter for the evaluation of IOL although relatively less reliable in MF, due to inflammatory state. In these patients, therefore, a more significant role is played by transferrin saturation (marker of free iron release, which causes organ damage), and it should be tested together with ferritin level trends at the onset of the disease.

The role of imaging techniques (T2-weighted MRI) in patients with MF is more controversial, due both to the difficulties in distinguishing between fibrosis-induced organ damage and iron-induced toxicity and because toxic iron-induced damage and exposure to ROS may clinically occur even before iron accumulation will be evident in the hepatocytes and myocardial cells.

### Iron Chelation Therapy and Myelofibrosis: Iron Chelation Response

#### Impact of Deferasirox Treatment in Reducing Ferritin and Inducing Erythroid Response in Transfusion-Dependent Myelofibrosis

Although the observation of potentially harmful iron-induced toxicity has suggested the utility of ICT in MF in the management of anemia, the clinical evidence regarding the use of iron-chelating agents is still limited. In fact, only single case reports regarding the use of ICT in MF were available until few years ago. Recently the results of two Italian multicenter studies became available with the following results (**Table 2**):

**Table 2:** Iron chelation efficacy and erythroid responses during deferasirox treatment in myelofibrosis patients.

Institution	Lombardy Hematological Network (68) (*)	Lazio Group (69) (**)
Evaluable patients	41/45 pts	47/48 pts
Median duration of treatment	17.2 months (IR 3–59.5)	27.6 months
Iron chelating response (ICR)	Ferritin <1,000 ng/ml or ≥50% decrease of baseline ferritin: 29.3% (12 pts) No response: 70.7% (29 pts)	Global response: 41% (19 pts) of which: Ferritin <500 ng/ml: 5 pts Ferritin <1,000 ng/ml: 11 pts ≥50% decrease of baseline ferritin but with ferritin level >1,000 ng/ml: 3 pts No response: 59% (28 pts)
Erythroid responses (ER) Transfusion independence ↑Hb ≥1,5 g/dl or decrease ≥50% of transfusional rate No response	17% (7 pts) 26.9% (11 pts) 56.1% (23 pts)	12,8% 6.4% 80.8%
Median time to ER	2.4 months (IR 1–8.5)	6.3 months (IR 4.3–12.1)

(*) starting dose 750 mg/day (10 mg/kg/day); (**): Starting dose 20 mg/kg in 23 patients, 15 mg/kg in 20 patients, and 10 mg/kg in 5 patients.

IR, interquartile; pts, patients.

- 45 patients with primary MF or Post Polycythemia Vera MF (PPV-MF)/Post Essential Thrombocythemia MF (PET-MF) treated between 2010 and 2018 in the Lombardy Hematological Network (IRON-M Study) (68)
- 48 patients with primary MF or PPV-MF/PET-MF treated by the Lazio Group (69)

These two studies report the impact of treatment with deferasirox in terms of induction of iron chelation response (ICR) and erythroid response. Based on these data, an ICR was obtained with deferasirox in approximately 30–40% of patients with MF, whereas complete or partial hematological response (usually not accompanied by an increase in platelets and neutrophils) was observed in 20–40% of cases.

The results show that in both studies, patients who obtained an ICR experienced a progressive reduction in ferritin levels at 6, 12, and 18 months, compared to non-responders (68). As regards the identification of factors predictive of an ICR to deferasirox, in the study by Elli et al. (68), patients who obtained efficacious ICR presented statistically lower ferritin levels at baseline than non-responders. Similarly, the median number of prior transfusions and the duration of the history of transfusion dependence were statistically lower/shorter amongst responders. These data highlight that an earlier access to iron chelation therapy constitutes an advantage in terms of efficacious iron chelation. On the other hand, other than the statistically significant relationship between the achievement of ICR and the obtainment of hematological improvement, it was not possible to identify factors predictive of hematological response (68).

The achievement of ICR, defined both by reduction in ferritin levels and by prolonged OS, are closely related in patients with MF treated with deferasirox (69): 2-years OS after the start of treatment was 100% in patients who responded to the ICT, compared to 70% in patients who did not experience a reduction in ferritin levels (68) (95% CI: 46.5–84.8%, p=0.007). In patients who presented a reduction in ferritin levels, median survival from the start of therapy with deferasirox was 61.0 months (95% CI: 18.4–103.6) compared to 15.8 months (95% CI: 5.9–25.6) in patients who did not respond to the ICT (p=0.001) (69). Therefore, by reducing the IOL, treatment with deferasirox seems to provide a significant advantage in terms of the survival in patients with MF. The rate of leukemia evolution or progression disease seems to be lower in ICR group, with a better 2-year leukemia free survival (LFS, p=0.039) (68), but this result needs to be confirmed in a larger and prospective cohort.

#### Patients with MF in Whom There is an Indication for Initiating Iron Chelation Therapy

The data currently available allow us to identify the eligible patient profile for ICT, for subjects with a life expectancy >6 months:

1. Patients with anemia not treated with ruxolitinib, when they become transfusion-dependent
2. Patients with transfusion-dependent anemia and significant symptoms and/or splenomegaly who start treatment with ruxolitinib, whose IOL is already significant at the start of therapy and will probably increase after the administration of ruxolitinib itself, as transfusion needs will increase
3. Transfusion-dependent anemic patients with an indication for HSCT, who require ICT in preparation for the transplant procedure

On the contrary, in non-anemic MF patients and who develop transfusion dependency in the early months after starting of ruxolitinib, the administration of an iron chelator may not be necessary due to the likelihood of Hb level recovery.

#### When a Patient With MF Treated With Deferasirox Must Be Considered a Responder

ICR to deferasirox is usually evaluated by taking into account ferritin value trends compared to the baseline. In the absence of standardized response criteria, ferritin levels <1,000 ng/ml or <500 ng/ml can be considered indicative of the achievement of ICR, as can a ≥50% decrease in ferritin levels compared to the baseline, regardless of the final levels.

As mentioned previously, given the inflammatory nature of the condition, although on the one hand these values are sufficient for MDS, they would appear to be less conclusive in patients with MF. Transferrin saturation rate could represent a more reliable marker of chelation efficacy useful also as markers of iron toxicity and ROS-induced damage; a threshold of 70% might be considered for this evaluation.

#### Safety Profile of Deferasirox in Myelofibrosis

Treatment with deferasirox in patients with MF can be associated with the onset of extra-hematological adverse events (AEs), which are primarily constituted by renal impairment: 17.7% (70), hepatic impairment: 8.9% (68), gastrointestinal symptoms: 11.1–21.4% (68, 69), and skin reactions: 6.7–7.2% (68, 69). Overall, a definitive discontinuation of ICT secondary to toxicity of grade ≥2 of DFX was reported in about a quarter of patients. Of note, no difference in term of number of AEs was seen between ICR and no ICR group; among the patients achieving ICR, no grade 3–4 toxicity was reported (68).

### Therapies for Myelofibrosis and Iron Chelation

#### Clinical Evidence Regarding Ruxolitinib-Deferasirox Combinations

In recent years the advent of the specific JAK-1/JAK-2 inhibitor ruxolitinib represented the only real innovation in the treatment of MF. The use of concomitant therapy with deferasirox in MF patients treated with ruxolitinib would appear based on a clinical need and a strong biological rationale (both drugs work synergically by reducing the oxidative damage in the bone marrow and interacting with the NF-kB and JAK-STAT pathways). The limited evidence currently available (70) suggests that this combination is feasible in terms of tolerability and efficacious in reducing iron overload.

### Iron-Induced Toxicity, Transfusion Requirements, and Iron Chelation Therapy in Patients With Aplastic Anemia and Other Chronic Anemias

#### Utility of Iron Chelation in Aplastic Anemia

Aplastic anemia (AA), a rare blood disorder, is considered the paradigm of bone marrow failure (BMF) syndromes. The disease is caused by an aberrant immune response, which causes the oligoclonal expansion of cytotoxic T cells, resulting in the destruction of haemopoietic stem cells (71). Clinically, it results in different grade pancytopenia (AA classified as “severe,” “very severe,” or “not severe” depending on bone marrow cellularity and peripheral cytopenia status).

Current treatment for AA (indicated in cases that are severe, very severe, or become transfusion-dependent) is based on allogeneic stem cell transplantation (in eligible patients) and on immunosuppressive therapy [cyclosporine, anti-thymocyte globulin (ATG)]. Patients who are not eligible due to old age or comorbidities and those who do not obtain clinical response can be managed with supportive care that includes the use of growth factors, antibiotic treatments, and transfusion therapy (54).

IOL, defined as a ferritin value >1,000 ng/ml, is present in almost 20% of patients who receive transfusions and in 6% of non-transfused patients (72). In a *post-hoc* analysis of the EPIC study, patients with AA were seen to have had an average of between 8 and 15 transfusion sessions in the previous year, and their median ferritin levels were 3,600–3,700 ng/ml (73). Using imaging techniques (MRI), IOL was observed in the liver in 76.4% of patients (including AA) and in the heart in 19.2% (74). The presence of an IOL in patients with severe AA reduces the efficacy of the immunosuppressive therapy and increases the risk of a relapse, confirming that it is a negative prognostic factor for the outcome of these patients (75).

Therefore, in AA there is a significant incidence of IOL, and its negative impact on clinical outcomes constitutes the rationale for using ICT in this setting. Furthermore, high ferritin levels might have a negative impact on the response to therapy and on the risk of toxicity in patients with AA undergoing a HSCT (76).

At the current time, there are no specific standardized criteria for starting ICT in patients with AA. The serum ferritin threshold value >1,000 ng/ml can be used as a rule of thumb (75, 77–80).

The EPIC prospective study evaluated the efficacy and safety of ICT with deferasirox in 116 patients with AA (74). The analysis showed that therapy with deferasirox efficaciously reduced median serum ferritin levels: this efficacy was associated with the transfusional iron load and the dose of deferasirox, with higher doses of medicinal product needed to reduce the iron load in subjects with a high intake (79).

In patients with AA, as in other blood disorders, IOL is associated with the observation of hematological response in patients that became transfusion-independent (73). In an Italian multicenter prospective study (81), hematological responses were observed with transfusion independence, associated with a significant increase in platelets, in a cohort of eight patients with AA treated with deferasirox in a real-life setting.

#### Other Types of Anemia in Which Iron Overload Occurs Regardless of the Transfusion Load

IOL is a common complication in patients with congenital hemolytic anemias (CHAs), a heterogeneous group of disorders that include hereditary spherocytosis, G6PD deficiency, and pyruvate-kinase (PK) deficiency. These patients can experience an IOL regardless of transfusion therapy due to the hyperhemolysis caused by the hereditary deficiency, as demonstrated by MRI evaluation that showed IOL also reported in patients with ferritin levels <1,000 ng/ml (82). In patients with PK deficiency who do not have regular transfusions, a total prevalence of IOL (based on ferritin values) of 38% was observed, which increases (to 82%) when MRI is used as the detection technique.

In an analysis conducted on a population of 37 cases of CHAs (26), the presence of IOL was seen to be common in patients with congenital hemolytic anemias, especially in those with PK deficiency and with congenital dyserythropoietic anemia type II (CDAII), who presented particularly high ferritin and transferrin saturation levels, even regardless of transfusion dependency. Moreover, hepatic iron overload (LIC) was observed in patients with not excessively high ferritin levels also in this study.

A specific sub-analysis of the EPIC study on a total population of 57 patients including 23 cases of hemolytic anemia (of which five cases of PK deficiency, two cases of congenital erythropoietic protoporphyria, and one patient with hereditary hemolytic anemia) showed efficacy of deferasirox in decreasing median serum ferritin levels (−617 ng/ml at 1 year), confirming the durable response after 1 year of treatment, regardless of the underlying pathogenic mechanisms (83).

## Management of Side Effects and Comorbidities

### Frailty of Elderly Hematological Oncology Patients and Comorbidities

When considering a new treatment approach, it is important to consider the patient age, which is often high, and the likely presence of comorbidities; the latter must not be seen as a contraindication for ICT, rather a supplementary factor to be taken into consideration in order to support its indication. Iron-mediated toxicity caused by excess free iron can even worsen an existing dysfunction or result in the emergence of concomitant subclinical conditions.

### Impact of Iron-Induced Toxicity on Renal Function

Impaired renal function is closely correlated to the age of the patient; over 40 years of age, 2/3 of the population present a progressive decrease in glomerular filtration rate (especially in males) (84). Changes in renal function are therefore more common in elderly patients and cardiac, hepatic or metabolic comorbidities (85).

The concomitant presence of anemia, hypoxia, and iron overload may impair the kidneys, especially in the tubular region. Moreover, these factors can cause oxidative stress in the tubular cells responsible for the resorption of water and solutes, which have a consistent ATP-dependent metabolic activity, with consequent histological and functional damage that, in turn, results in increased urinary excretion of certain solutes, including albumin, proteins, potassium, uric acid, calcium, phosphorus and bicarbonate. This excretion inevitably leads to conditions such as metabolic acidosis, bone changes, and kidney stones (85–91).

Chelation agents such as deferoxamine and deferasirox are thought to be responsible for the onset of renal changes and, in a small percentage of cases, also for severe forms of acute renal impairment, although the pathogenetic mechanism of this effect is still unclear. The onset of Fanconi syndrome (generalized proximal convoluted tubule defect) has also been observed, associated, not necessarily concomitantly, with polyuria, proteinuria, hypouricemia, hypopotassemia, hypercalciuria, hypophosphatemia, metabolic acidosis (caused by decreased bicarbonate resorption), and bone demineralization (due to the buffer effect of bone that binds with the acids) (92, 93).

As demonstrated by pivotal clinical studies on the use of deferasirox (94–96), up to one-third of cases present renal function changes, most of which are mild and within range of normality. These changes should not be considered clinically significant, as they are slight, non-progressive fluctuations in creatinine levels, with a less than 33% increase compared to the baseline, reversible (in most cases), and transient. In the GIMEMA MDS0306 study (31) just one in 15 cases of renal function changes was grade 3. In most cases, the change in renal function resolves spontaneously.

IOL leads to kidney injury secondary to oxidative stress. However, iron constitutes an important co-factor in prostaglandin synthesis and in the production of ATP in the kidney tubule cells by the mitochondria (85). This results in decreased sodium and chlorine resorption localized in the proximal convoluted tubules, which in turn causes increased resorption of both electrolytes in the distal portion of the tubules (macula densa) anatomically adjacent to the glomerulus. This process transmits a vasoconstriction signal to the afferent arteriole of the glomerulus, triggering a reduction in glomerular filtration (tubuloglomerular feedback).

The decreased levels of prostaglandins, caused by the lack of production, induce vasoconstriction on the afferent arteriole that leads to the impairment of glomerular blood flow. This results in a reduction in glomerular filtration rate that, together with renal flow, normalizes when treatment is discontinued, even after 2 years of treatment (97, 98). When the benefits of the treatment are considered to outweigh the risks associated with the increase in creatinine (which is non-progressive and lower than 33% compared to the baseline) and/or proteinuria (values lower than 0.5 g/24 h), it is possible to continue treatment with deferasirox without dose adjustments.

All patients should then undergo (also with the aid of a nephrology specialist) renal function laboratory monitoring, including assessments of glomerular and tubular function.

When changes in renal function in patients with chronic anemia eligible for ICT are observed, it is necessary to consider all factors potentially responsible for renal injury as

- anemia;
- IOL;
- viral infections (HBV, HCV, HIV);
- comorbidities (heart and liver disease, diabetes mellitus, arterial hypertension, dyslipidemia);
- smoking habits; and
- patients, especially elderly ones, who are co-administered nephrotoxic drugs (non-steroidal analgesics, antibiotics, proton pump inhibitors, and allopurinol) (99, 100).

#### How to Monitor Renal Function at the Start of and During Therapy With Deferasirox

In patients with IOL (regardless of treatment with deferasirox), it is important to monitor renal function by taking into consideration factors such as:

- albumin-to-creatinine ratio,
- protein-to-creatinine ratio.

Both tests are simple to perform on the first morning urine sample of the day, and the results correlate with those over 24 h.

Other important, but less extensively studied, aspects are

- tubular function and
- venous blood gas analysis (to identify states of metabolic acidosis).

**Table 3** provides a possible monitoring schedule during treatment with deferasirox.

**Table 3:** Optimum renal monitoring schedule.

Tests	Baseline	First months	First 6 months	Every 6 months
Nephrology consultation	X			
General functional markers:				
▪ Creatinine	X	X	X	X
▪ Urinalysis	X	X (every week for first month)	X	X
▪ Cystatin C	X			X
▪ Urinary protein/creatinine ratio (mg/g)	X		X (every month for 6 months)	X
Markers of glomerular function:	X		X (every month for 6 months)	X
▪ Urinary albumin/creatinine ratio (mg/g)				X
Markers of tubular function:				
▪ Urinary Beta2-microglobulin/creatinine ratio (mcg/g)	X			X
▪ Urinary calcium/creatinine ratio (mg/g)	X			X
▪ Urinary phosphorus/creatinine ratio (mg/g)	X			X
▪ Urinary NGAL/creatinine ratio (mcg/g)	X			X
▪ Venous blood gas analysis	X			X

### Hepatic Comorbidities and Iron Chelation

The presence of hepatic comorbidities needs to be carefully considered for appropriate multidisciplinary management: the free iron can lead to functional changes in liver parenchyma that, if not appropriately managed, can result in cirrhosis and liver cancer.

Treatment with deferasirox is not recommended in patients with severe hepatic impairment (Child-Pugh class C), whereas for Child-Pugh class B patients, who present moderate hepatic impairment, the dose should be considerably decreased and progressively increased if needed. However, baseline hypertransaminase should not be considered an absolute contraindication to start therapy, as it may be sign of iron-induced toxicity that will be reduced by ICT (101, 102). For a correct management of hepatic enzymes, it is important to perform baseline tests and constant monitoring of AST/ALT, bilirubin and alkaline phosphatase, and of the transfusion burden (main cause of transaminase elevation, which conditions the adjustment of the dose of iron chelator). In order to identify the cause of the liver disease, it is therefore essential to work closely with a hepatology specialist.

#### The Role of T2-Weighted MRI in Establishing the Relationship Between Liver Disease and Iron Overload

MRI and the transverse relaxation rate (R2*) are non-invasive methods for a quantitative assessment of the iron content of almost the whole organ, unlike biopsy. While these methods are well established in thalassemic patients, the use of MRI in transfusion-dependent patients is increasing in recent years (103, 104). This can be limited by the fact that most of transfusion-dependent patients are elderly ones, in whom the organ damage may occur even before iron accumulates in liver and heart cells; this may correlate with higher mortality in this group of patients (105). A recently published Italian cooperative study (MIOMED) well demonstrated the fundamental role of T2*-MRI in detecting IOL: amongst 50 patients affected by low-/intermediate-1 MDS, 13 showed a hepatic and 13 a cardiac IOL. The hepatic involvement was significantly conditioned by transfusion load that, at the contrary, did not influence the cardiac status. When patients were prospectively evaluated, after 12 months two (non-chelated) developed hepatic IO and other two heart damage (106).

### Patients on Polytherapy: The Most Common Pharmacological Interactions With Deferasirox

#### The Clinical Role of and Rationale for Plasma Deferasirox Testing

From a pharmacokinetic standpoint, deferasirox is characterized by a somewhat reduced volume of distribution, circulation that is 99% bound to serum proteins, especially albumin, metabolism that occurs largely by UGT1A1-mediated glucuronidation (and 8% in the liver by cytochromes), and fecal elimination (107).

It has been observed (108, 109) that the plasma levels of deferasirox are closely influenced by different polymorphisms, such as the levels of enzymes involved in the metabolism of the medicinal product and of efflux pumps (UTG1A1, CYP1A1, CYP1A2, CYP2D6, ABCG2), which therefore directly influence the safety and efficacy profile of the medicinal product. For example, toxicity in patients with homozygosity for UGT1A1*6 is some 14 times greater than wild-type patients.

Given the number of genes involved, typing is challenging in clinical practice; this difficulty also regards hepatic transporters like MRP2, involved in the biliary excretion of the medicinal product, whose polymorphisms can increase hepatotoxicity seven-fold (108).

One solution could be to test plasma deferasirox levels, although this approach is not currently used in clinical practice.

#### Products That May Interact With Deferasirox

For patients with MF and MDS, concomitant use of medicinal products is an important issue. As mentioned previously, great care is required when using medicinal products such as non-steroidal anti-inflammatory drugs (NSAIDs) and angiotensin converting enzyme (ACE) inhibitors, whereas there is no literature evidence available for other interactions, such as deferasirox + erythropoietin, lenalidomide, and demethylating agents (azacitidine and decitabine).

In literature, there are comforting data regarding the synergistic use of deferasirox with other medicinal products: for example, the use of this treatment with anti-leukemia therapy would appear to improve its efficacy.

In patients with MDS on treatment with demethylating agents, which have an epigenetic effect, deferasirox has been reported to be additive or synergistic with azacitidine and decitabine (an association that reduces proliferation, increases apoptosis, and halts the cell cycle in leukemia cell lines and has a more potent effect than the individual products) (110). Moreover, treatment with deferasirox results in the upregulation of the HJV gene that encodes for hemojuvelin, suggesting that deferasirox might play a role in the demethylation of certain agents together with decitabine (110).

The other therapeutic effects of deferasirox regard the upregulation of TP53 oncosuppressor gene expression (111) and reduction in the outflow proteins, such as the P-glycoprotein, the levels of which are increased by iron through the production of ROS, which decreases after treatment with deferasirox and increases the efficacy of chemotherapy agents (112).

Synergistic use of deferasirox and eltrombopag increases the mobilization of cell iron by the latter, which acts as a shuttle that removes the iron present in the tissues and rapidly transfers it to the former (113, 114).

In patients undergoing HSCT, special attention must be dedicated to busulfan and specific inhibitors (ibrutinib, ruxolitinib), as concomitant use with deferasirox can increase the plasma levels of these agents.

## Conclusions

In patients with MF and MDS, but also with AA and hemolytic anemias, the prognosis is not merely associated with individual factors such as age, comorbidities, or underlying condition, but also with complications associated with the degree of cytopenia present (severity of anemia, hemorrhages, and infections) and the treatment that they receive (blood transfusions, allogeneic transplants, etc.). In these patients, any therapeutic interventions should also face not only the management of comorbidities but also complications caused by the evolution of disease and adverse effects of primary treatments.

The excess iron, associated with direct and indirect toxicity on the various tissues, leads to a worsening in age-related comorbidities, ultimately resulting in cumulative organ damage. Iron-induced toxicity also affects the evolution of the MDS or MF, by increasing cellular genomic instability and altering the bone marrow stroma through the oxidative stress, thus favoring progression to acute leukemia (15, 65).

The performance of a multidisciplinary evaluation of patients treated with deferasirox, at baseline and during follow-up, makes it possible to improve the therapeutic approach and optimally manage elderly patients with MF and MDS, consequently avoiding the need to discontinue specific therapy and reducing the risk of developing organ damage.

## Author Contributions

GP, SG, RR, and RL conceived the manuscript and prepared the first draft. All authors critically revised the article for important intellectual content. All authors gave final approval of the version to be published and agreed to be accountable for all aspects of the work in ensuring that questions related to the accuracy or integrity of any part of the work are appropriately investigated and resolved. All authors contributed to the article and approved the submitted version.

## Funding

Medical writing assistance for the preparation of this article was funded by Novartis Pharma, Italy. The funder was not involved in the writing of this article or the decision to submit it for publication.

## Conflict of Interest

GP has received honoraria and/or served on the scientific advisory boards for Abbvie, Amgen, AOP, AstraZeneca, BMS Celgene, Janssen, and Novartis. WB: Consultant Novartis. EE: Participation to advisory board Novartis. CF: Novartis: advisory committees, speaker fees; Celgene: research funding, advisory committees, speaker fees; Takeda: consultancy. PM has received honoraria and/or served on the scientific advisory boards for Celgene, Janssen, Takeda, Bristol-Myers Squibb, Amgen, Novartis, Gilead, Jazz, Sanofi, Abbvie, Incyte, and Glaxo-Smith-Kline.

The remaining authors declare that the research was conducted in the absence of any commercial or financial relationships that could be construed as a potential conflict of interest.

## Publisher’s Note

All claims expressed in this article are solely those of the authors and do not necessarily represent those of their affiliated organizations, or those of the publisher, the editors and the reviewers. Any product that may be evaluated in this article, or claim that may be made by its manufacturer, is not guaranteed or endorsed by the publisher.
